# Modeling the Effect of Streetscape Environment on Crime Using Street View Images and Interpretable Machine-Learning Technique

**DOI:** 10.3390/ijerph192113833

**Published:** 2022-10-24

**Authors:** Huafang Xie, Lin Liu, Han Yue

**Affiliations:** 1 Center of Geoinformatics for Public Security, School of Geography and Remote Sensing, Guangzhou University, Guangzhou 510006, China; 2 Department of Geography, University of Cincinnati, Cincinnati, OH 45221, USA

**Keywords:** street view images, machine-learning, XGBoost, SHAP, interpretability

## Abstract

Street crime is a common social problem that threatens the security of people’s lives and property. Understanding the influencing mechanisms of street crime is an essential precondition for formulating crime prevention strategies. Widespread concern has contributed to the development of streetscape environment features as they can significantly affect the occurrence of street crime. Emerging street view images are a low-cost and highly accessible data source. On the other hand, machine-learning models such as XGBoost (eXtreme Gradient Boosting) usually have higher fitting accuracies than those of linear regression models. Therefore, they are popular for modeling the relationships between crime and related impact factors. However, due to the “black box” characteristic, researchers are unable to understand how each variable contributes to the occurrence of crime. Existing research mainly focuses on the independent impacts of streetscape environment features on street crime, but not on the interaction effects between these features and the community socioeconomic conditions and their local variations. In order to address the above limitations, this study first combines street view images, an objective detection network, and a semantic segmentation network to extract a systematic measurement of the streetscape environment. Then, controlling for socioeconomic factors, we adopted the XGBoost model to fit the relationships between streetscape environment features and street crime at the street segment level. Moreover, we used the SHAP (Shapley additive explanation) framework, a post-hoc machine-learning explainer, to explain the results of the XGBoost model. The results demonstrate that, from a global perspective, the number of people on the street, extracted from street view images, has the most significant impact on street property crime among all the street view variables. The local interpretability of the SHAP explainer demonstrates that a particular variable has different effects on street crime at different street segments. The nonlinear associations between streetscape environment features and street crime, as well as the interaction effects of different streetscape environment features are discussed. The positive effect of the number of pedestrians on street crime increases with the length of the street segment and the number of crime generators. The combination of street view images and interpretable machine-learning techniques is helpful in better accurately understanding the complex relationships between the streetscape environment and street crime. Furthermore, the readily comprehensible results can offer a reference for formulating crime prevention strategies.

## 1. Introduction

### 1.1. Street Crime and Streetscape Environment

With the development and integration of environmental criminology and geographic information science, scholars have gradually begun incorporating built-in environmental factors into crime analysis. A series of theories have been proposed in the process, such as rational choice theory, routine activity theory, CPTED, etc. These theories emphasize the importance of the built environment in crime research from different perspectives. The rational choice theory argues that certain built-in environmental features can influence offenders’ behaviors by affecting their perceptions of risk and cost [1,2], which in turn affects the spatial distributions of crime. The routine activity theory claims that the built-in environmental features can impact the spatial-temporal overlap of offenders, potential victims, and ineffective supervisors, which in turn affects the occurrence of crime [3]. The crime pattern theory holds that particular places or facilities can act as crime ‘generators’ or ‘attractors’ by gathering potential victims and offenders throughout their daily activities [4,5]. Such places include schools, parks, liquor stores, banks, stations, etc. [4,6,7,8,9]. Based on the above theories, CPTED is mainly concerned with how to prevent crime by altering the built-in environment. It advocates that proper environmental design and reasonable and effective utilization of the built environment can improve the order of the environment and enhance residents’ sense of security so as to prevent crime [10].

Various crime theories claim that environmental features have a direct impact on the occurrence of street crimes and that streets are where street crimes occur. Therefore, understanding the criminal impact of the street environment and spatial change is essential in reducing the risk of crime. Among other factors affecting the street environment, the presence of people on the street has been demonstrated to be an important factor affecting street crime. However, different scholars have different opinions regarding the effect of people on the street on street crime. On the one hand, crowds on the street provide suitable targets for street crimes such as pickpocketing. Therefore, streets with more people are usually associated with higher street crime rates because the plentiful crime opportunities attract potential offenders [11,12,13]. On the other hand, some scholars argue that people on the street can act as guardians and deter potential offenders from committing crimes. Therefore, the number of people on the street should be inversely related to crime because an increase in the number of people means an increase in guardianship level [14]. Thus, the exact role of the crowd in street crimes requires further examination.

The physical elements of the streetscape also have an important influence on street crime. For example, the sidewalk in an area indicates that there may be pedestrians who are potential criminal targets. Previous research demonstrated that theft crimes occur more often in areas with sidewalks than in areas without them. The reason may be that many offenders commit thefts by trailing behind pedestrians [15]. Walls and fences could control the accessibility of the area, increasing the difficulty of escaping after committing crimes. Therefore, the existence of walls and fences could somewhat help inhibit the occurrence of crime [1,16,17]. The appearance of street lights [18] or surveillance cameras [19] reflects the surveillance level of a territory. A strong sense of territory can increase the probability of criminals being discovered or caught, thus deterring rational offenders [20,21,22]. Green elements such as trees and grass can facilitate people’s outdoor activities and thus promote informal monitoring, thereby restraining the occurrence of crimes. However, thick shrubs may obstruct people’s lines of sight and provide hiding places for offenders; therefore, areas with shrubs have potential crime risks [1,23,24,25]. A holistic investigation of these features and their possible interactions remains unanswered.

Although researchers have begun to include environmental variables when conducting crime analyses, there are still challenges in measuring environmental features, especially characteristics of the micro-streetscape environment. Traditional methods of measuring environmental characteristics include questionnaires [26], field collections [27,28,29], etc. These measurement methods can extract the micro-built environmental features as fine as possible. However, they are only suitable for small research areas due to the enormous workload, time-consuming, and sizeable manual errors. Although remote sensing images can offer large-scale environmental information, they cannot capture the street-level microenvironment from the perspective of pedestrians. In order to solve these data gaps, street view imagery is increasingly being used to obtain large-scale detailed environmental features.

Street view imagery is a type of big geographic data that emerged with the development of artificial intelligence. Through street view images, researchers can obtain a 360° view of street-level environmental information from the pedestrian perspective without leaving home; based on this view they can measure the street-level microenvironment efficiently. For example, Ewing [30] took photos of 48 commercial streets in the United States, established an evaluation index system, and used the expert scoring method to measure the quality of street space. He et al. [31] used Google Street View imagery for environmental auditing. They collected the microscopic physical environment features of the streets around the residential area (such as graffiti on the walls, dilapidated houses, abandoned vehicles, etc.) by manually viewing online street view images. Curtis [32] and Marco [33] also used similar methods to extract physical environment information from Google Street View imagery. However, extracting street environment features from street view images by artificial methods is also time-consuming, labor-intensive, and highly subjective; therefore, it is only suitable for small-area research. Additionally, the results can be easily affected by human subjectivity.

As computer vision technology matured, researchers gradually combined deep learning image extraction methods with street view images to extract large-scale street microenvironment features. For example, Hipp [1] used deep learning methods to extract pixel proportions of streetscape elements such as buildings, people, sidewalks, vehicles, fences, and walls from Google Street View images. Based on this data, they analyzed the relationship between different dimensions of environmental characteristics and different types of crime. Using similar methods, Amiruzzaman [34] extracted elements such as roads, sky, and vegetation in high- and low-crime areas to study the relationship between street visual environment characteristics and crime. Their analysis showed that the visual features of the street environment could distinguish areas with different crime rates. Khorshidi [35] and Sytsma [36] also used similar methods to extract street environment information from street view images to study the relationships between street features and crime. However, existing studies mainly applied a single semantic segmentation method to extract street environment elements from street view images. These studies ignored the different appearances of different elements in street view images, which may lead to unreliable results. Additionally, previous research mainly focuses on the impact of individual street environmental characteristics on crime, ignoring the nonlinearity of street environmental characteristics and the interaction effect between streetscape features and community socioeconomic conditions.

### 1.2. Interpretable Machine-Learning Technique

Machine-learning is the process by which computer programs learn from experience or training data to optimize model performances [37]. It can incorporate various datasets and rely on the computer’s automatic training and analysis capabilities to obtain the optimal model without requiring researchers to set specific parameters. Additionally, fitting models through machine-learning algorithms often produces better results than traditional regression methods [38]. At present, machine-learning technology has been widely used in many fields, including health research [39,40], weather forecasting [41], power systems [42], etc. Various machine-learning algorithms are emerging along with the development of artificial intelligence. Commonly used machine-learning algorithms include support vector machine [43], K-nearest neighbors [44], Random Forest [45], and Gradient Boosting [46]. However, a severe disadvantage of these machine-learning algorithms is their non-transparency and non-interpretability. This makes it difficult for users to understand the model’s results [47], thus making it hard to spot errors and improve model performances [48]. Because of these flaws, some scholars have questioned the results of machine-learning models. For instance, Das and Rad suggested [49] not blindly trusting the results of models with high prediction accuracies.

When using machine-learning methods for crime analysis and modeling, these “black box” models will prevent researchers from understanding why such results appear and which variables significantly affect the occurrence of crime. At the same time, the results cannot provide substantive guidance for police departments to make crime prevention and control strategies. Therefore, there is an urgent need to find effective methods to address machine-learning models’ transparency and interpretability issues.

According to existing research, there are two ways to improve the transparency and interpretability of machine-learning models: ante-hoc techniques and post-hoc techniques. Ante-hoc techniques can generate explanations for results from when the model starts training; therefore, they are suitable for explaining transparent models [50]. Post-hoc techniques generate explanations after model training and are suitable for non-transparent models such as support vector machines and neural networks [50]. In addition, post-hoc interpretability includes global interpretability and local interpretability. Global interpretability can explain the “black box” models and help researchers understand how the results are generated and which elements play more critical roles in the entire model [51]. Local interpretability can help in the understanding of the decision-making process of a particular input sample [52].

Based on routine activity theory and CPTED theory, this study collected streetscape environmental features from large-scale street view images and applied an interpretable machine-learning approach to examine the influence of streetscape elements on street crime. Specifically, we constructed a Faster R-CNN object detection network to detect discrete elements with distinct outlines, such as people. We constructed a PSPNet semantic segmentation network to detect streetscape features without definitive shapes, such as the sky and roads. A series of socioeconomic and facility characteristics were accounted for as control variables. Based on this, we trained an XGBoost model to extract the effect of the streetscape environment on street crime. The SHAP interpreter was then used to understand the model results. Specifically, this study revealed the contributions of streetscape environment features to street crime at both global and local levels. More importantly, we examined the interaction between street environment features and community socioeconomic attributes.

## 2. Study Area, Data, and Method

### 2.1. Study Area

We conducted this study in the main urban area of ZG city, a megacity along the southeastern coast of China. The complex population structure, built environment, and frequent socioeconomic activities in this place attract nearly one-third of the city’s crimes. The research unit is the street segment, with 2930 street segments in the study area. As a developed urban area, the coverage of the Baidu street view (BSV) images in this place is complete and dense. Additionally, since the ZG city is located in a subtropical area, the street environment is not affected too much by seasonal changes. Therefore, this study extracted streetscape environmental elements from Baidu Street View images.

### 2.2. Data

#### 2.2.1. Crime Data

**Figure 1:**
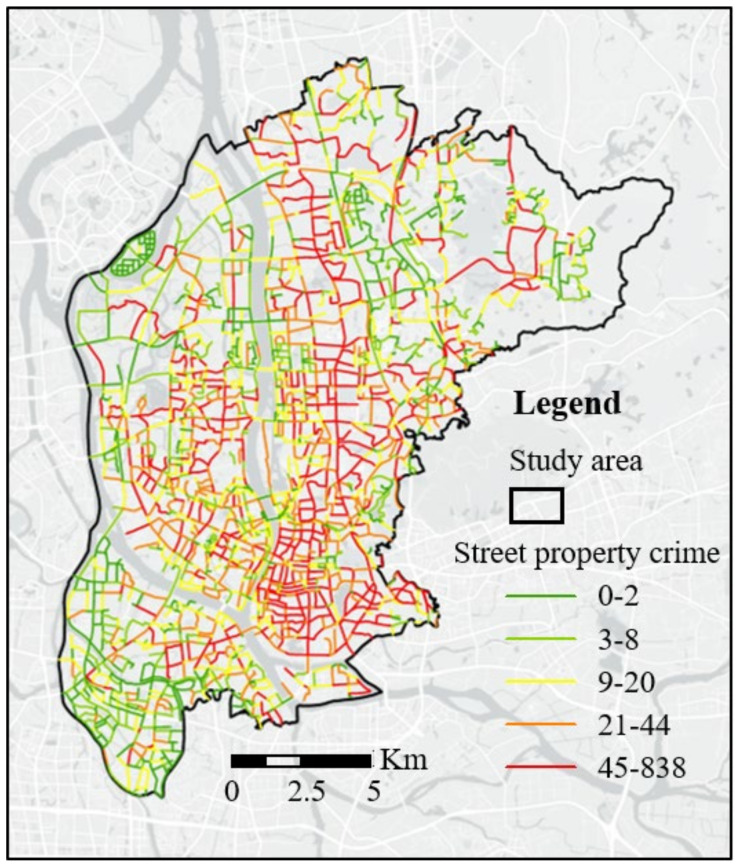
The spatial distribution of the number of street property crimes at the street segment level.

The type of crime studied in this paper is street property crime, including theft in public places, pickpocketing, and robbery. We obtained three-year (2017–2019) street property crime data in the study area from the ZG city police department. There were 84025 street property crimes in the study area in this period. We projected each crime to its nearest street segment and calculated the number of crimes on each street segment. Figure 1 presents the spatial distribution of the number of street property crimes at the street segment level.

#### 2.2.2. Streetscape Environment Variables

**Figure 2:**
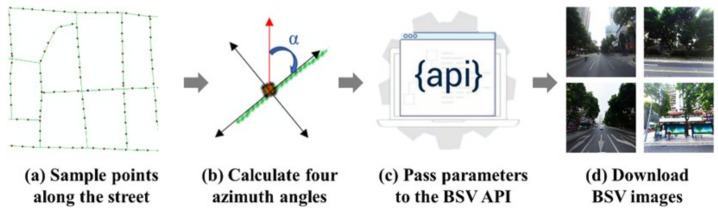
Steps of downloading BSV images.

With the development of artificial intelligence technology, more and more scholars combine machine-learning methods and street view images to fulfill large-scale audits of the street environment. This study used the BSV images as the data source for the streetscape environment variables. Two deep learning methods, object detection and semantic segmentation, were adopted to extract street microenvironment elements from these images. Firstly, we generated a sampling point every 10 m along the street segment to obtain the street environment features as ultimately and uniformly as possible (see Figure 2a). Then we calculated the azimuth angles in four directions, two parallel to the streets and the other perpendicular to the streets (Figure 2b). Using the locations of the sampling points and the azimuth angles as input parameters, we downloaded four BSV images at each sampling point through the BSV API (Figure 2c,d). To ensure the street environment variables matched the crime data, we only kept the BSV images captured between 2016 and 2019. Finally, 129,719 sampling points were created and 518,876 street-view images were downloaded in the study area. The average file size of the street view images was about 500 kb per image. Therefore, the total file size of all the street view images was about 247 GB.

**Figure 3:**
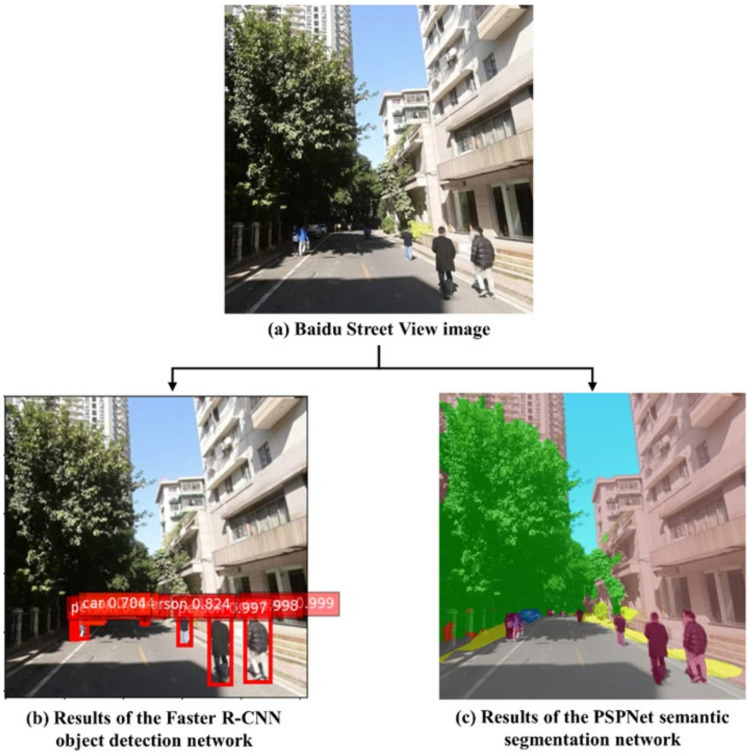
An example of (**a**) a Baidu Street View image, (**b**) the results of the Faster R-CNN object detection network, and (**c**) the results of the PSPNet semantic segmentation network.

We used the Faster R-CNN object detection network to extract discrete elements with distinct outlines in each BSV image, such as people and streetlamps, see Figure 3b. Then, the total numbers at the street segment level were calculated using Formula (1). We used the PSPNet semantic segmentation network to extract the elements without definitive shapes in the images, such as the sky, sidewalk, building, wall, fence, tree, grass, and roads (see Figure 3c). Then we used Formula (2) to calculate the average pixel proportions of these elements at the street segment level. Based on these results, we calculated the number of discrete elements and the average proportions of the pixels of the elements without definitive shapes on each street segment.

(1) $$\begin{array}{c} Total\;number\;of\;discrete\;elements\\ with\;distinct\;outlines \end{array}=\sum_{i=1}^{n}\sum_{j=1}^{4}Count_{Image_{ij}\left(x\right)}$$

(2) $$\begin{array}{c} Average\;pixel\;proportion\;of\;elements\\ without\;distinct\;outlines \end{array}=\frac{\sum_{i=1}^{n}\sum_{j=1}^{4}Pixel_{Image_{ij}\left(x\right)}}{\sum_{i=1}^{n}\sum_{j=1}^{4}Pixel_{Image_{ij}}}$$

where $Count_{Image_{ij}\left(x\right)}$ indicates the number of discrete element *x* with distinct outlines in the *j*th street view image at the *i*th sampling point; $Pixel_{Image_{ij}\left(x\right)}$ represents the number of pixels of element *x* without distinct outlines in the *j*th street view image at the *i*th sampling point, and $Pixel_{Image_{ij}}$ represents the total number of pixels in the image.

Based on the CPTED, the routine activity theory, and previous research experience, we selected 11 streetscape environment variables: pedestrians, sidewalks, buildings, walk suitability, walls, fences, sense of enclosure, street lamps, trees, grass, and green rate. Among them, pedestrians, sidewalks, buildings, walls, fences, street lamps, and grass were directly extracted from BSV images. Three composite variables, walk-suitability, sense-enclosure, and green rate, were calculated based on values of individual elements extracted from BSV images. According to the CPTED theory, certain environmental features, such as walls and fences, can act as access control measures and inhibit the occurrence of crimes [1,16,17]. Better lighting conditions can enhance regional territorial surveillance and deter potential criminals [20,21,22]. Green spaces such as trees and grassland can facilitate residents’ outdoor activities and thus promote informal monitoring of the area, thereby retraining the occurrence of crimes [1,23,24,25]. In addition, people on the street may act as potential victims, and this factor is usually associated with high crime rates [11,12]. Apart from pedestrians directly related to the people on the street, this study also considered the proportions of sidewalks and buildings, as these two elements are also associated with human activity [1].

We also calculated three composite variables from the individual variables extracted from the street view images. Walk suitability was used to characterize the degree to which the street environment supports pedestrian walking activities. A good walking environment is beneficial to increasing the level of natural surveillance in the environment [14]. Walk suitability was calculated using Formula (3), where the sidewalk represents walkable areas and the road represents drivable areas. The fence could create safe conditions for pedestrians by separating sidewalks and roads. Overall, walk suitability represents the degree to which the street environment supports walking. As represented by Formula (4), sense of enclosure refers to the proportional relationship between the vertical elements like buildings and trees and horizontal elements like roads, sidewalks, and fences along both sides of the street, which can affect the pedestrian safety. A street segment with a high sense of enclosure offers pedestrians a high sense of security, which promotes people’s outdoor activities [53]. The green rate, calculated by Formula (5), refers to the proportion of green areas within the range of vision. It is also associated with people’s sense of security [23].

(3) $$Walk\;suitability=\frac{\frac{1}{n}\sum_{i=1}^{n}Sidewalk_{i}+\frac{1}{n}\sum_{i=1}^{n}Fence_{i}}{\frac{1}{n}\sum_{i=1}^{n}Road_{i}}$$

(4) $$Sense\;of\;enclosure=\frac{\frac{1}{n}\sum_{i=1}^{n}Building_{i}+\frac{1}{n}\sum_{i=1}^{n}Tree_{i}}{\frac{1}{n}\sum_{i=1}^{n}Road_{i}+\frac{1}{n}\sum_{i=1}^{n}Sidewalk_{i}+\frac{1}{n}\sum_{i=1}^{n}Fence_{i}}$$

(5) $$Green\;rate=\frac{1}{n}\sum_{i=1}^{n}Tree_{i}+\frac{1}{n}\sum_{i=1}^{n}Grass_{i}$$

where $Sidewalk_{i}$, $Fence_{i}$, $Road_{i}$, $Building_{i}$,$\;Tree_{i}$, and $Grass_{i}$ represent the pixel proportion of sidewalks, fences, roads, buildings, trees, and grass, respectively, using Formula (2).

**Figure 4:**
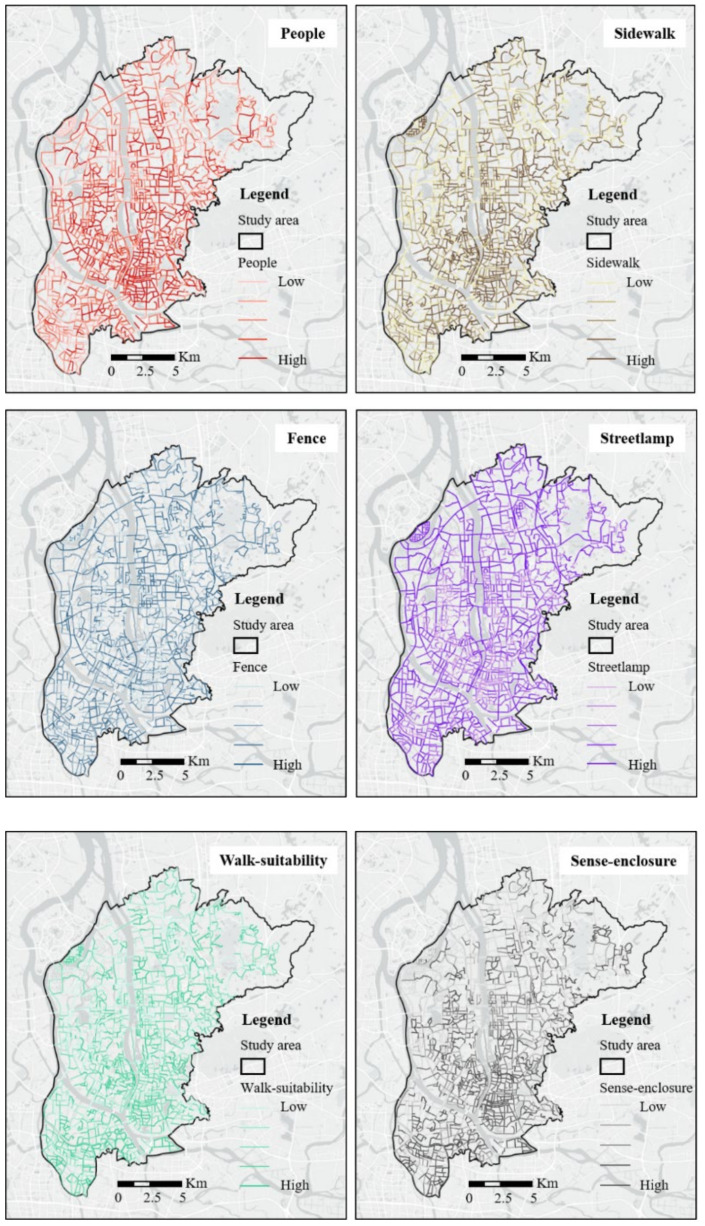
Examples of spatial distributions of street view variables.

Figure 4 shows some examples of spatial distributions of street view variables at the street segment level.

#### 2.2.3. Socioeconomic and Facility Variables

Many studies have shown that the accumulation of crimes in space relates to socioeconomic and facility features. Based on the routine activity theory and the crime pattern theory, this study controlled the effects of socioeconomic and facility features. Specifically, we first constructed a concentrated disadvantage index by combining three variables from the census data: the proportion of undergraduate people, the proportion of young people (aged 19–45 years), and the proportion of floating people. The raw socioeconomic disadvantage variable is a community feature, so we used the sample average method [1,54] to compute it at the street segment level. The adjusted Herfindahl-Hirschman index, calculated using Formula (6), was used to calculate the land-use mix of the street segments’ surrounding areas [17].

(6) $$POI\;Mixture=1-\sum_{i=1}^{n}Proportion_{i}^{2}$$

where $Proportion_{i}$ represents the proportion of the number of POIs of *i*th type. The values of the POI mixture are between zero and one and a larger value means a more diverse land use.

The street segment length was included to account for the possibility that more crimes may occur on longer streets. The density of street networks surrounding the street segment was obtained to proxy regional transportation convenience. In addition, crime generators and attractors could provide crime opportunities for potential offenders. Based on the existing literature, five types of POIs (catering, shopping, accommodation, bus stops, and subway stations) were included as crime generators [55,56]. Bars, internet cafes, game halls, and other sports and leisure POIs were included as crime attractors. When calculating the number of crime generators and crime attractors on a street segment, only POIs within 100 m of the street segment are included [57]. The descriptive statistics of the dependent and independent variables used in this study are listed in Table 1.

### 2.3. Methods

#### 2.3.1. XGBoost

Chen and Guestrin proposed the XGBoost (eXtreme Gradient Boosting) model in 2016 [58]. XGBoost is an ensemble model used for classification and regression tasks; the model is an integrated gradient-boosting decision tree constructed by combining multiple weak decision trees. The XGBoost model enhances its classification and regression capabilities by iteratively minimizing the output value of the objective loss function. A function is constructed at each iteration to fit the differences between actual and predicted values. As XGBoost has high prediction accuracy and a fast processing speed with a low computational cost and complexity, it is commonly used in various fields of study. Tuning and finding optimal parameters can help maximize the performance of the XGBoost model [58]; Parameter tuning is also crucial for preventing overfitting. However, manually doing the parameter tuning process can take considerable time and resources. Grid search and random search are two popular automatic parameter-tuning methods. Grid search is slow but can traverse the entire search space; random search is fast but may miss important points in the search space. As an alternative, the Bayesian optimization method proposed by Snoek [59] can avoid the above problems. The main idea of the Bayesian parameter optimization method is that, given the objective optimization function, it updates the posterior distribution of the objective function by continuously adding sample points until the posterior distribution fits the actual distribution. The objective optimization function refers to a generalized function; we only need to specify the input and output as the functions’ internal structures and mathematical properties do not need to be known. Simply put, the Bayesian parameter optimization method adjusts the current parameters based on the information generated from the last iteration. As the Bayesian optimization method is effective, efficient, and easy to use, this study used it to generate the optimal hyperparameters for the XGBoost model.

#### 2.3.2. SHAP

Generally speaking, ensemble machine-learning models such as XGBoost have high accuracy and have achieved great success in many fields. However, as “black box” models, these methods have high internal complexity, making the established models lack interpretability, and people cannot understand them intuitively [60]. Fortunately, S.M. Lundberg and S.I. Lee [61] proposed the SHAP (SHapley Additive exPlanation) framework, which can be used to interpret the results of complex machine-learning models. As a classic post-hoc attribution interpretation framework, the SHAP framework interprets the results of machine-learning models by calculating the contribution of each feature after adding it to the models. Therefore, SHAP is a class of additive explanatory models, which treats each feature as a contributor, calculates its specific contribution value, and sums the contribution values of all features to generate the final prediction [62].

The core of the SHAP framework is the Shapley value proposed by L. Shapley in 1953, originally designed to solve the distribution equilibrium problem in cooperative game theory [63]. In the SHAP framework, a Shapley value represents the “weight” or “importance” of a particular feature when the model makes predictions about a particular data point. The Shapley values of different variables are additive. For each sample, the final predicted value equals the sum of the average predicted value and the Shapley values of all the explanatory variables. This characteristic ensures that the sum of the contribution values equals the final output value and eliminates the explanatory differences caused by the model structure. The feature importance generated by traditional methods, such as linear regression models, can only indicate the more important features. However, it is not clear how the feature affects the prediction results. However, SHAP can reveal how features affect the prediction results by differentiating the positive and negative effects. Based on this, SHAP applies to local and global interpretations [64]. For local interpretability, each feature has its own set of Shapley values. Therefore, it can help us interpret the contribution of each feature to each sample’s prediction value, which increases the transparency and reliability of the prediction model. The global interpretation concerns each feature’s overall importance: the average of the Shapley values of the specific variable in all samples. Since the SHAP explanation model is consistent with human intuition, it has become increasingly popular in explaining machine-learning models in recent years. This study analyzes the effect of the street view and control variables on street crime based on the SHAP framework and interprets the results based on relevant crime geography theories.

## 3. Results

This study used Python 3.8 to train the XGBoost model and then used the SHAP explainer to interpret the contributions of each variable to the dependent variable. The main optimal hyperparameters generated by cross-validation are as follows: learning_rate: 0.05, max_depth: 2, number_of_estimators: 500, min_child_weight: 20, gamma: 1.35, colsample_bytree: 0.95, subsample: 0.8. The model is of high accuracy, with an R-square value of 0.47. In the following sections, we introduce the results of the XGBoost model interpreted by the SHAP explainer.

### 3.1. Global Interpretability

This study used the SHAP interpreter to explain the results of the XGBoost model. Specifically, the contributions of explanatory variables can be interpreted at both global and local levels. The SHAP values represent the magnitudes and directions of the influences of independent variables on the dependent variable. The mean absolute SHAP values in all research units reflect the global interpretability of the XGBoost model, which allows us to understand the contribution of each independent variable as a whole [52].

**Figure 5:**
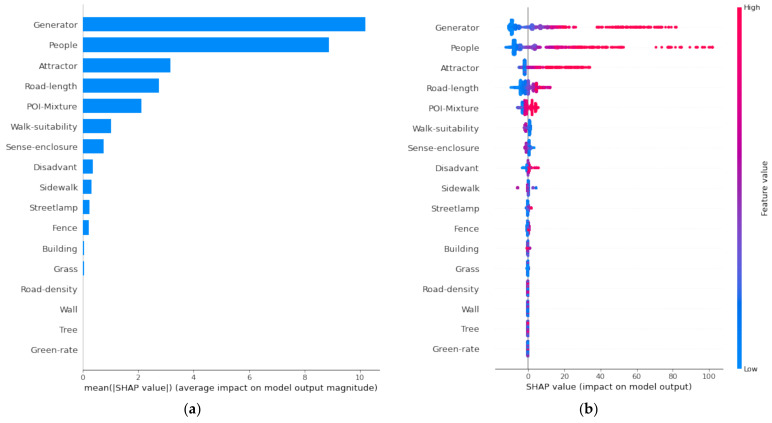
Interpreting the results of XGBoost using SHAP explainer at the global level. (**a**) Mean absolute SHAP values. (**b**) SHAP values of each sample.

Figure 5a shows the mean absolute SHAP values sorted in descending order. The lengths of the bars represent the average impacts on model output, which proxies the global influences of explanatory variables on the dependent variable. According to the results, the number of crime generators (Crime generator) and people on the street (People) have the largest averages of SHAP values. Therefore, from an overall perspective, these two factors have the most substantial explanatory power for the occurrence of street crime. The number of attractors (Crime attractors), the length of the street segment (Road-length), and the mixing degree of POIs (POI-Mixture) are three factors that are also very important for street crime, although the extent of their contribution is much smaller compared with the top two variables. In addition to the number of people on the street, walk suitability (Walk-suitability) and sense enclosure (Sense-enclosure) are the other two characteristics that have the most critical effect on street crime among the street view variables. As to the remaining street view variables, only the proportion of sidewalks (Sidewalk), the number of street lamps (Streetlamp), and the proportion of fences (Fence) also have apparent influences on street crime. The socioeconomic disadvantage in the areas surrounding the street segment (Social and economic disadvantage) also affects the occurrence of street crime. The other variables, such as the proportion of buildings (Building), the proportion of grass (Grass), the road density in the areas surrounding the street segment (Road-density), the proportion of walls (Wall), the proportion of trees (Tree), and the greening rate (Green-rate), do not have apparent influences on street crime.

In order to better understand how each independent variable affects the dependent variable, we plotted all the research samples’ feature values and corresponding SHAP values together in an image, as shown in Figure 5b. In the image, a point represents a research sample (a street segment in this study), and the color represents feature values, where red represents high values and blue represents low values. The *x*-axis shows the SHAP value, which is a proxy for the influences of the independent variables on the dependent variable. If the SHAP value is positive, there is a positive relationship between the independent and dependent variables, and a negative value indicates a negative relationship. The results presented in Figure 5b are consistent with those presented in Figure 5a. Figure 5b shows that the first line represents the number of crime generators (Generator). Street segments with more crime generators (represented symbolically by red points) also have higher SHAP values. In comparison, street segments with fewer crime generators (represented by blue points) have smaller SHAP values. That is to say, the number of crime generators not only has the most significant impact on street crime (Figure 5a), but this impact is also positive (Figure 5b). Similarly, the number of people on the street (Person) and the number of crime attractors (Attractor) also positively affect street crime. The effects of other features on street crime can be interpreted in the same way.

### 3.2. Local Interpretability

The total SHAP value (f(x)) of a street segment is equal to the baseline value (the mean of the total SHAP values of all street segments) plus the sum of the SHAP values of all independent variables. Suppose the total SHAP value of a street segment is greater or smaller than the baseline value, meaning that the contributions of the features of the street segment jointly increase or decrease the risk of crime. Different locations have different SHAP values for the same independent variable. The spatial variation reveals the different effects of local microenvironment features on different street segments.

**Figure 6:**
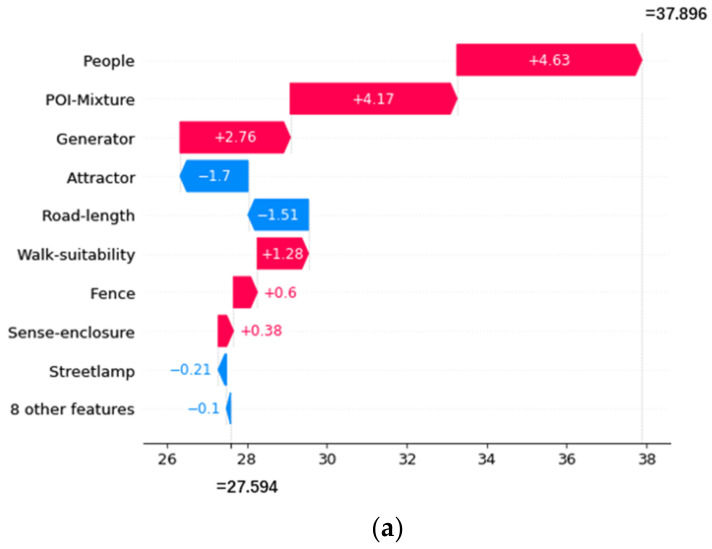

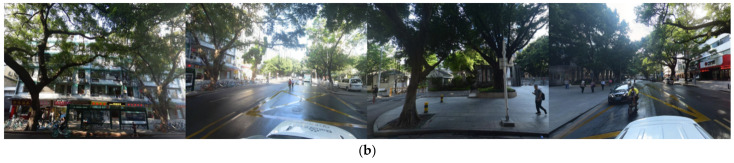
Local SHAP values of the features of the street segment with the highest frequency of street property crime. (**a**) Local SHAP values. (**b**) Examples of street-view images.

**Figure 7:**
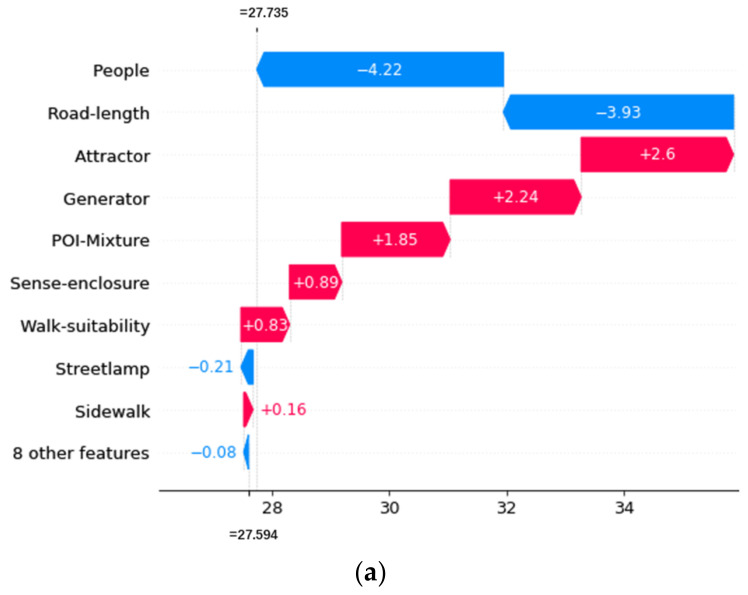

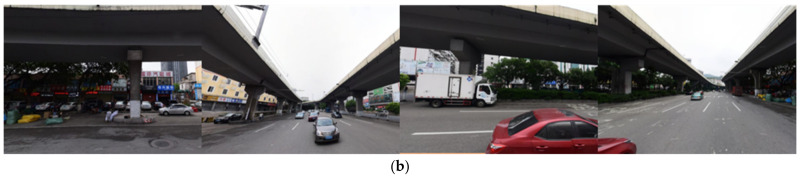
Local SHAP values of the features of the street segment with the mean frequency of street property crime. (**a**) Local SHAP values. (**b**) Examples of street-view images.

**Figure 8:**
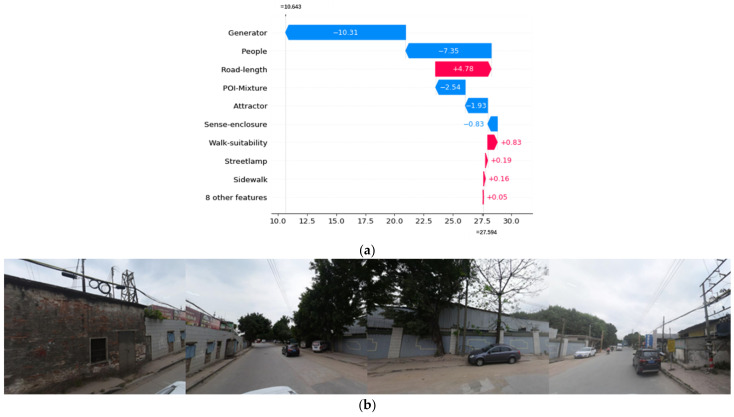
Local SHAP values of the features of the street segment with the smallest frequency of street property crime. (**a**) Local SHAP value. (**b**) Examples of street-view images.

Three street segments with high, mean, and low frequencies of street property crimes are selected as examples to explain the local effect of street environment features. The SHAP values of each feature are visualized as waterfall plots in the following pictures. As shown in Figure 6a, Figure 7a and Figure 8a, the red bars indicate positive influences of the variables, and the blue bars indicate negative influences. The lengths of the bars represent the absolute contributions of the variables, which are marked in the figures. Furthermore, we also presented real street view images of the street segments, as shown in Figure 6b, Figure 7b and Figure 8b.

It can be seen from Figure 6a that for the street segment with the highest crime frequency, the number of people on the street (People) has the most significant impact, with a SHAP value of 4.63. The mixing degree of POIs (POI-Mixture) and the number of crime generators (Generator) also have large and positive impacts on street property crime. That is to say, the high frequency of crime on this street segment is mainly caused by a large number of people, the complex land use conditions, and the dense crime generators. The environmental information reflected by the examples of the street view images on this street is consistent with the model results. As shown in Figure 6b, wide sidewalks are populated by pedestrians and cyclists, who are potential victims of street property crime. Moreover, the street is lined with numerous commercial facilities (crime generators and attractors) such as government offices and banks (crime attractors). In addition, tall trees and buildings stand on both sides of the street, which indicates high walk suitability. These environmental characteristics indicate that this street segment has specific social and economic services that attract a high latent intensity of social and economic activities. These streetscape environment features and complex land use patterns can attract many people to this street segment for daily activities. At the same time, wide sidewalks, tall trees, and buildings provide pedestrians with a comfortable walking environment, which enhances the willingness of pedestrians to walk or stay in this area. According to the crime pattern theory and the routine activity theory, this street segment is attractive to motivated offenders and is an ideal choice for street property crime.

As shown in Figure 7a, for the street segment with the mean crime frequency, the top three variables with the greatest impacts on street property crime are the number of people on the street (People), the length of the street segment (Road-length), and the number of crime attractors (Attractor). In addition, the effect of crime generators (Generator) is slightly less than that of crime attractors. The number of people on the street and the length of the street segment have negative impacts on street property crime, while the impact of crime attractors is positive. The results suggest that more people on the street may lead to fewer street property crimes. It is noteworthy that the relationship between the number of people and street property crime is different here compared to the street segment with the highest frequency of street property crime. The street view images of this street segment presented in Figure 7b can help in understanding the results. As can be seen from the images, the street segment is characterized by sparse crowds (representing potential victims or monitors), heavy traffic, and an abundance of commercial facilities (representing crime generators and attractors). The intensity of socioeconomic activity here is slightly lower than that of the street segment with the highest crime frequency. Although there are no large crowds on this street segment, the commercial shops here suggest potential socioeconomic activities in the area. Therefore, we believe that a limited number of people on this street segment will provide potential victims, thus creating a certain number of crime opportunities in the area. However, in this environment with limited population density, the effect of human surveillance may be more critical than the attracting effect. Together, these environmental characteristics contribute to the limited street crime intensity.

Figure 8a shows that for the street segment with the least crime frequency, the three variables that have the greatest impact on street property crime are the number of crime generators (Generator), the number of people on the street (People), and the length of the street segment (Road-length). Furthermore, the impacts of the number of crime generators and the number of people on the street are negative. As we can see from the street view images in Figure 8b, there are only a very small number of people and vehicles on the street. The houses on both sides of the street are dilapidated, indicating economic disadvantage. Combined with street view images, we can conclude that in areas with very few pedestrians and socioeconomic activities, the appearance of people here could play an inhibitory role in street crime. Generally, the above three samples of street segments have significant differences in socioeconomic and built-in environmental features, revealing that the contributions of variables are affected by the local microenvironment.

### 3.3. Nonlinearity and Interactivity

**Figure 9:**
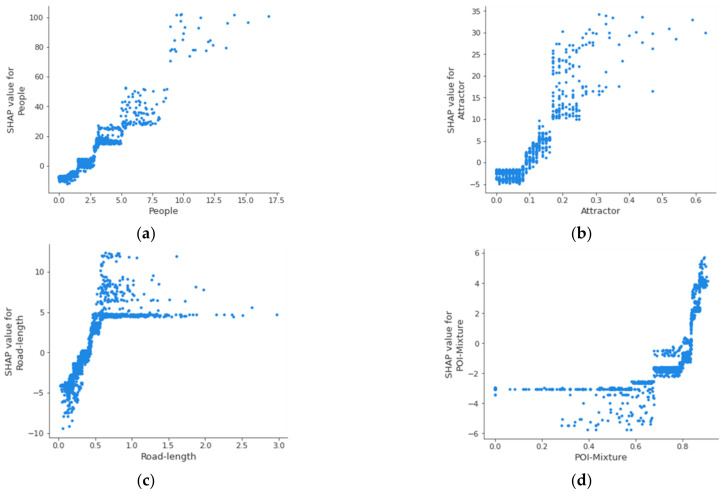
The nonlinear effect of explanatory variables on street property crime. (**a**) the number of people on the street, (**b**) the number of crime attractors, (**c**) the length of the street segment, (**d**) the mixture of POIs.

To observe the relationship between individual variables and street property crime, we plot Figure 9 based on the values of the variables (*x*-axis) and the SHAP values (*y*-axis). The four images demonstrate the positive nonlinear relationship between street property crime and the number of people on the street (Figure 9a), the number of crime attractors (Figure 9b), the length of the street segment (Figure 9c), and the mixed degree of POIs (Figure 9d). Specifically, Figure 9a–c show that larger values of the three variables (the number of people on the street, the number of crime attractors, and the street length) are correlated with higher SHAP values. However, when the variables grow to a certain extent, the growth of the SHAP values declines and approaches a steady state. The results suggest that a street segment will suffer from more street property crime if there are more people or more crime generators on the street segment or if the street segment itself is longer. Nevertheless, there is also a saturation effect. When the values of these variables increased to a certain extent, their effects on street property crime did not change significantly. In contrast, as shown in Figure 9d, the land use mixture of the areas surrounding the street segment (proxied by the mixture of POIs) has a stable and weak impact on street property crime when the degree of land use mixture is small. However, when the land-use mixture reaches a high level, its impact on street property crime increases rapidly. Therefore, since street property crime tends to occur on street segments with high land use mixture, and in these street segments, a slight increase in land use mixture can lead to a significant increase in the risk of street property crime. The land use mixture reflects the complexity of land use and population composition in a region. Furthermore, it identifies the region’s potential victims and supervision level. Thus, areas with high degrees of land use mixture have higher crime risks.

**Figure 10:**
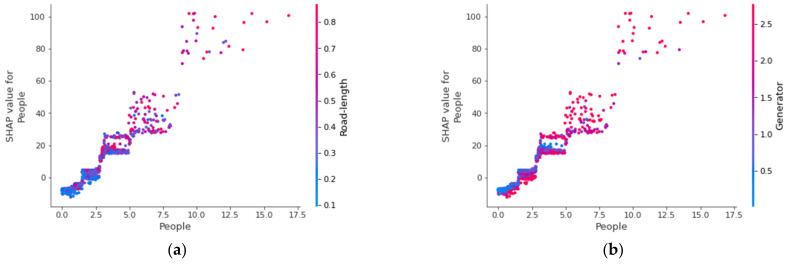

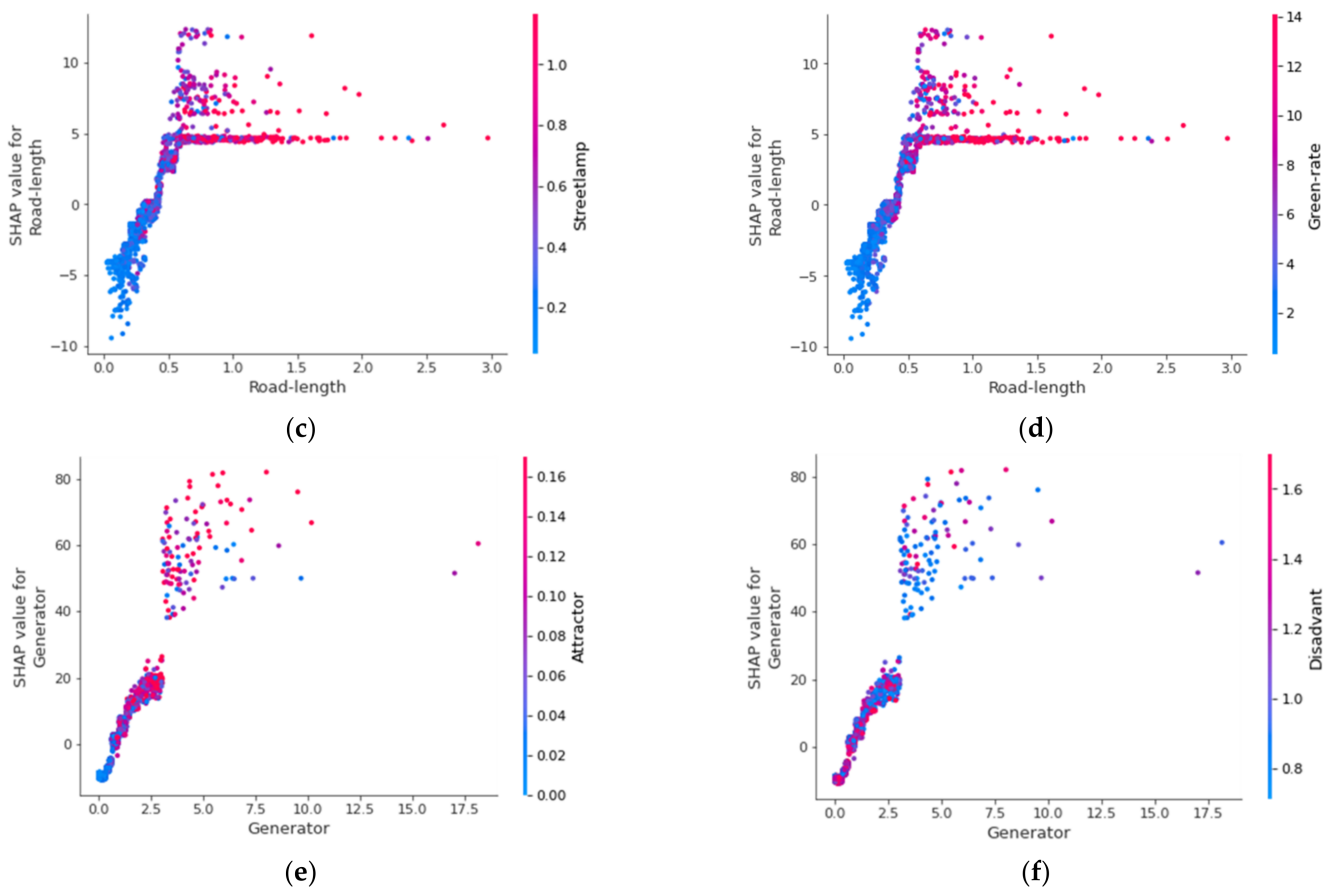
The interaction effect between different variables. (**a**) the number of people on the street and the length of the street segment, (**b**) the number of people on the street and crime generators, (**c**) the length of the street segment and the number of street lights, (**d**) the length of the street segment and the green rate, (**e**) the number of crime generators and crime attractors, (**f**) the number of crime generators and the community socioeconomical disadvantage.

The influence of a streetscape feature on street crime is not fixed but varies with other streetscapes or socioeconomic variables. Figure 10 reveals such interaction effects between different variables. The *x*-axis in each image represents the values of an explanatory variable, and the *y*-axis on the left represents the SHAP values corresponding to this variable. The *y*-axis on the right represents the values of another explanatory variable. Each dot stands for a research sample, and the color represents the variable’s value, as indicated in the *y*-axis on the right.

Figure 10a shows that street segments with more pedestrians also have longer lengths. For such street segments, the SHAP value of pedestrians increases gradually with the length of the street segment. This indicates a positive interaction effect between the number of pedestrians and the length of the street segments. The positive effect of pedestrians on street property crime increases with the length of the street segment. Therefore, when controlling for the number of pedestrians, longer street segments tend to experience more street property crimes. Moreover, the positive interaction effect gradually slows down as the number of pedestrians increases. The results reveal a nonlinear trend in the impact of the number of pedestrians on crime. Similarly, Figure 10b shows a positive interaction effect between the number of pedestrians and crime generators. The positive effect of the number of pedestrians on street crime increases with the number of crime generators and then gradually slows down. Therefore, the number of people on the street and the number of crime generators promote each other’s positive effects on street crime. Figure 10c,d presents a positive interaction effect between road length, the number of street lamps, and the green rate. The positive effect of road length on street property crime becomes stronger when the number of street lamps and the green rate both increase. Therefore, controlling for the length of the street segment, more street lamps, or a higher green rate results in a higher risk of street crime.

As shown in Figure 10e, there is a positive interaction effect between the number of crime generators and crime attractors. That is to say, the street segment with more crime generators also has more crime attractors. The SHAP values of crime generators on street segments with large numbers of crime generators are high. The results suggest that street segments with more crime generators and crime attractors may experience higher rates of street property crime. In contrast, there is a negative interaction between the number of crime generators and socioeconomic disadvantage, as presented in Figure 10f. Generally, more crime generators are located in street segments with lower socioeconomic disadvantages. Therefore, street segments with more crime generators and low levels of socioeconomic disadvantage are more vulnerable to street property crime. It is readily comprehensible that street segments with more crime generators or lower levels of socioeconomic disadvantage may have more potential victims and opportunities for crime.

## 4. Discussion

In this study, street-view images were used as the data source for the street environment. Two deep learning methods of object detection and semantic segmentation were combined to quantitatively measure the street environment features. In this way, we achieved a refined description of the street microenvironment. Controlling for the impact of socioeconomic factors, we modeled the influences of the streetscape environment on street property crime using the XGBoost model. XGBoost is a machine-learning algorithm with better a fitting accuracy than traditional regression models [65,66]. However, as a “black box” model, the implementation process of this type of machine-learning algorithm is not transparent, and we cannot intuitively know the contributions of different variables to the model results. Therefore, it cannot provide substantive guidance for formulating crime prevention and control strategies. To address this issue, we used the SHAP interpreter to solve the interpretability problem and analyze the model results at both global and local levels. Furthermore, we also analyzed the nonlinear relationships between the streetscape environment and street property crime and the interaction effects between different variables.

To interpret the contributions of different variables from a global perspective, we calculated the mean absolute SHAP values of the independent variables. The ranking results show that the number of crime generators, the number of people on the street, and the number of crime attractors have the most substantial global explanatory power for street property crime. In particular, the mean absolute SHAP values of the first two variables are 10.1838 and 8.8830, respectively, which are much larger than the other variables. Therefore, street segments with more crime generators or people on the street are more vulnerable to street property crime.

Our results are consistent with existing crime geography theories and experimental studies. According to the crime pattern theory, certain places, such as restaurants, shopping centers, bus stops, and subway stations, are usually located in the routine activity spaces of both offenders and potential victims. These places are called “crime generators” in crime geography. Motivated offenders usually seek opportunities to commit crimes during routine activities [67]. Therefore, street segments with more crime generators usually have higher crime risks. Andresen et al. [68] obtained results similar to ours, showing that crime generators on the street, such as bus stops and commercial retail outlets, positively influence street crime. The routine activity theory is closely associated with the crime pattern theory. According to the routine activity theory, people’s daily activities affect the spatiotemporal collection of intentional offenders, potential victims, and the absence of guardians, which in turn affects the temporal and spatial patterns of crime [69]. Moreover, street property crimes such as theft, pickpocketing, and robbery usually happen with concealment. Crowded streets are ideal places for committing crimes because the large crowds in these places not only provide numerous targets for offenders but also cover for offenders and avoid attracting too much attention. As such, streets with dense populations are ideal locations for street property crime. The study by Yue et al. also proves this viewpoint [17]. Another type of place that has attracted wide attention to the crime pattern theory is the crime attractor. These places easily attract criminals to commit crimes due to many criminal opportunities, such as frequent cash transactions [67,70]. Crime attractors can also lead to the spatial accumulation of criminal activities. Therefore, street segments with many crime attractors or crime generators have higher incidences of street crime.

As a vital streetscape variable, the number of people on the street has also been proven to significantly influence the occurrence of street property crime in other studies [1,17]. On the one hand, people’s routine activities affect the spatiotemporal patterns of crime [69]. On the other hand, street property crimes, such as theft, pickpocketing, and robbery, have concealment characteristics. Not only do crowds provide offenders with numerous opportunities to commit crimes, but they also provide cover for offenders by avoiding attracting too much attention. Therefore, motivated criminals tend to commit crimes in crowded places. That is to say, streets with dense populations are ideal locations for street property crime. Other street view variables, such as walk suitability, sense of enclosure, the proportion of sidewalks, and the proportion of street lamps, also affect street property crime, but their global effects are not strong.

Local interpretability analysis of the model results demonstrates that the local contributions of individual variables are not spatially fixed but are significantly related to the local area’s socioeconomic and built-in environmental conditions. For example, local analysis results show that the number of people affects street crime differently in street segments with different crime risks. The results are consistent with crime theory and existing empirical research. According to the rational choice theory, offenders choose appropriate places to commit crimes after rationally evaluating cost, risk, and benefit [71]. Generally speaking, offenders of property crimes tend to commit crimes in places with high social and economic vitality [4], dense crowds [72], and a lack of effective supervision [73]. For the street segments with high crime intensities, banks, government offices, bus stops, and numerous commercial shops on both sides attract intense socioeconomic activity. Offenders find these socioeconomic activities attractive enough as they have the potential to meet their income expectations. At the same time, the spacious sidewalks and tall trees or buildings beside the streets provide a comfortable environment for pedestrians to walk [74], which in turn facilitates nearby residents to carry out outdoor activities [75]. For criminals, more people means more opportunities for crime. Furthermore, dense crowds can also cover offenders’ criminal behavior, reducing the risk of being discovered and arrested. These characteristics together lead to the high crime intensity on such street segments. There are usually few socioeconomic activities for the street segments with low crime intensities, and the mode of land use is single. Such economically disadvantaged areas are not attractive to street property offenders motivated mainly by monetary income. According to the routine activity theory, a person can act not only as a crime victim but also as a monitor [76,77]. In areas with relatively low socioeconomic vitality and few people, the informal surveillance role of people on the street surpasses the victim role. Therefore, such street segments are the least susceptible to street property crime. Similar to the number of people, there are also local differences in the contribution of crime generators and land use mix.

Testing for nonlinear effects of variable contributions shows that the contributions of several variables to street property crime are not monotonous. Taking the number of people on the street as an example, the size of the population in the environment directly reflects the environment’s vitality. Therefore, more people on the street means more crime opportunities. Nevertheless, there may be a saturation effect when the population size is large enough. The presence of many people on the street has not resulted in increased street property crimes. The reason may be that, as the number of people on the street increases, the number of potential victims also increases, but so does the number of monitors. Excessive crowds may have a deterring effect on potential offenders. The effects of the number of crime generators and street segment length on street crime follow similar patterns to the number of people on the street. The mixture of POIs characterizes the land-use complexity of the street segment’s surrounding areas. However, its nonlinear relationship with street crime differs from the aforementioned three variables. Only when the mixed degree of POIs reaches a relatively large value will it cause a significant increase in the number of street property crimes. The complexity of land use in an area is directly related to its social and economic vibrancy. The single land utilization in the street segment areas will limit socioeconomic vitality. The simple population composition will reduce the size of crowds, which reduces the possibility of attracting street property offenders.

Street property crime is not only affected by the value of individual variables but also by the interaction effect between different variables. The reason is that each variable does not act independently on the occurrence of street property crimes, but the effect is affected by other variables. The results show a positive interaction effect between the number of people and road length, the green rate, and the number of crime generators. Road length positively interacts with the number of street lamps and the green rate. The results reveal that the impact of the street environment on street property crime is not independent but is affected by socioeconomic attributes. Specifically, the extent and direction of the effect of a streetscape feature on street crime are not the same on different street segments but differ with the socioeconomic features of the surrounding communities. Taking the number of people and the length of the road as an example, both positively correlate with street property crime. Their positive interaction effect further reveals that the longer the street segment, the stronger the impact of the number of people on the amount of street property crime. Similarly, the number of people also positively interacts with the number of crime generators. Therefore, a street segment with more crime generators indicates a stronger positive relationship between the number of people and street property crime. According to the crime pattern theory, crime generators such as shopping malls and supermarkets are important elements of people’s routine activity spaces. These places usually attract a large number of potential victims and motivated criminals at the same time, which creates conditions for crimes [67]. A larger number of people on the street could increase the risk of street crime, and the risk is further increased if there are more crime generators on the street. Therefore, different variables jointly shape the spatial pattern of street property crimes.

## 5. Conclusions

In this study, the street view images provide the data source for quantitatively describing the streetscape environment. The XGBoost model can accurately fit the relationship between streetscape environmental characteristics and street crime. The SHAP explainer offers an intuitive interpretation of the model results. The combination of street view images, the XGBoost model, and the SHAP explainer in this research is helpful in understanding more deeply the associations between street crime and features of the streetscape environment. The findings may help in the development of strategies to prevent and control crime. However, there are still some shortcomings in this study. Firstly, additional data sources can be included to enable a more comprehensive measure of the streetscape environment. Current methods cannot extract small objects such as garbage and broken windows from street view images. However, because these objects are essential indicators of physical disorder, future research should try to devise a way of extracting these objects from street-view images. Secondly, the samples used in this study were all obtained from the main urban area of ZG City. Follow-up research should concern the relationship between street crime and the streetscape environment in different areas, such as the suburbs. Because there is a significant difference between the socioeconomic and built-in environment backgrounds of urban and suburban areas, the effect of the streetscape environment on street crime may also be different.

## Figures and Tables

**Table 1:** Descriptive statistics of variables.

Category		Name	Unit	Mean	SD	Min	Max
Dependent variables		Street property crime		28.68	48.92	0	838
Independent variables	Streetscape variables	People	per 100	1.46	1.74	0	16.83
		Sidewalk	%	3.48	2.29	0	15.80
		Building	%	19.70	12.70	0	64.70
		Walk suitability	-	0.64	17.75	0	959.50
		Wall	%	2.88	3.50	0	38.20
		Fence	%	0.91	0.90	0	10.90
		Sense of enclosure	-	3.11	27.90	0	901.90
		Street lamp	per 100	0.41	0.43	0	4.92
		Tree	%	17.40	10.10	0	51.40
		Grass	%	0.65	1.37	0	13.70
		Green rate	-	4.78	4.90	0	55.01
	Socioeconomic and facility variables	Social and economic disadvantage	-	1.19	0.31	0.45	2.21
		Crime generator	per 100	0.81	1.12	0	18.17
		Crime attractor	per 100	0.05	0.06	0	0.63
		POI mixture	-	0.76	0.16	0	0.90
		Road density	km/km^2^	26.56	9.63	5.21	66.41
		Road length	km	0.37	0.27	0.02	2.97
